# Ultrafast and stable planar photodetector based on SnS_2_ nanosheets/perovskite structure

**DOI:** 10.1038/s41598-021-98788-x

**Published:** 2021-09-29

**Authors:** Leyla Shooshtari, Ali Esfandiar, Yasin Orooji, Mahmoud Samadpour, Reza Rahighi

**Affiliations:** 1 Department of Physics, Sharif University of Technology, P.O. Box 11155-9161, Tehran, Iran; 2 College of Materials Science and Engineering, Nanjing Forestry University, Nanjing, 210037 People’s Republic of China; 3 Department of Physics, K.N. Toosi University of Technology, 15418-49611 Tehran, Iran; 4 SKKU Advanced Institute of Nano-Technology (SAINT), Sungkyunkwan University, Seobu-ro, Jangan-gu, 2066, Suwon, Gyeonggi-do 16419 Republic of Korea

**Keywords:** Materials science, Nanoscience and technology, Optics and photonics, Physics

## Abstract

Two-dimensional (2D) transition metal dichalcogenides are promising candidates of photodetectors where they are commonly grown parallel to the substrate due to their 2D characteristics in micrometer scales from exfoliation of bulk crystals or through high temperature chemical vapor deposition (CVD) methods. In this study, semi-hexagonal vertical nanosheets of SnS_2_ layered have been fabricated on FTO substrate without using Sn source through CVD method at relatively low temperature (500 °C). Due to exceptional band alignment of triple cation lead perovskite (TCLP) with semi-hexagonal SnS_2_ nanosheets, an improved photodetector has been fabricated. This type of photodetectors fabricated through lithography-free and electrodes metallization free approach with remarkable fast response (20.7 µs/31.4 µs as rising /falling times), showed high photoresponsivity, external quantum efficiency and detectivity of 1.84 AW^−1^, 513% and 1.69 × 10^11^, respectively under illumination of incident light with wavelength of 445 nm. The stability of the photodetectors has been studied utilizing a protective PMMA layer on the perovskite layer in 100% humidity. The introduced growth and fabrication process of the planar photodetector, including one/two dimensional interface through the edges/basal planes of layered materials with perovskite film, paves a way for the large scale, cost-effective and high-performance optoelectronic devices.

## Introduction

The broadband photodetectors which are commonly used in signal optoelectronic device systems, demand high performance, lower preparation costs and good commercialization prospect^1^. There are few reports on continually operating in UV to IR of photodetectors based on InSe^2^, GaSe^3^, and GaS^4^ as the novel nano-devices, but the challenge to access low-cost fabrication method, friendly environmental, high performance and wide range response photodetector at room temperature condition still exists^5^. Currently, several 2D layered semiconductors such as TMDs can be achieved through top-down (e.g. mechanical exfoliation) and bottom-up (e.g. CVD) processes^6^. In a viable development point of view, earth-abundant 2D materials are more crucial for prevalent use in the novel optoelectronic devices^7–9^. In this regard, the SnS_2_ crystal, belonging to IV-VIA group with electronic/optical band gap of about 2.1–2.31 eV displays a proper candidate for using in nano-electronic/optoelectronic and photoelectron chemistry^10,11^. Moreover, achieving vertical TMD structures could be the promising geometry in devices due to including more active surface area and highly active edge-sites of the layers rather than the basal planes^12^. To attain a vertical device, getting the growth of free-standing vertical nanosheets on the planer substrate is the major bottleneck, because unlike the 1D semiconductor nanowires, generally, the preferable growth direction of the 2D crystals is parallel to the substrates^13,14^. In this order, P.A Hu et al. reported a modified CVD method to grow vertically free-standing SnS_2_ nanosheets on the Si/SiO_2_ and fluorine-doped tin oxide (FTO) flat substrates using highly reactive precursors of tin chloride pentahydrate^15^.

In another side, utilizing various photo-absorbing materials such as quantum dots(QDs)^16^, carbon nanotubes^17^, graphene^18^, and organic molecules^19^ in a desired energy band alignment with the other components, could improve the absorption of the incident light and enhance the electron–hole charge carries generation in electronic devices. Moreover, organo/inorgano halide perovskite (ABX_3_), owing to have large absorption coefficient and long-life photon-generated carriers are potent absorption materials in optoelectronic application^20–22^. The MAPbI_3_ (methylammonium lead tri-iodide) as the most popular type of perovskite shows phase transition under different conditions^23^. It has been recognized that partial substitution of methylammonium with formamidinium cation and mixing the bromide and iodide respectively as organic and halide part of the perovskite, along with adding a little amount of Cesium (Cs) leads to better stability and performance of perovskite material^24,25^. The lower trap density as 1.75 × 10^13^ cm^−3^ for three cationic lead iodide based perovskite on formamidinium and suitable electron mobility in the range of 54.6–157.1 cm^2^s^−1^ V^−1^ makes this type of perovskite the strong light absorbing layer in the photodetector devices^26^.

Although solar-cell like multilayers are capable to be planar photodetector, these structures often suffer from low sensitivity and huge electrical hysteresis^27^. Applying the interface perovskite layers with other functional materials such as TMDs^28^, carbon based material^29^, and metal oxide^30^, is powerful approach to improve the requirements of photodetectors. In this research, the vertical hexagonal SnS_2_ nanosheets on FTO substrate has been achieved using a modified CVD method without using extra tin (Sn) source at a relatively low temperature of 500 °C, which inherent Sn source in the FTO substrate plays a significant role to prepare these structures. To avoide the complexity of device fabrication based on TMDs, such as mechanical/chemical exfoliations, flakes transferring and lithography steps along with high vacuum deposition for electrode preparation, the planar TMD photodetector design based on patterned FTO has been presented here. Also, to improve photodetection, designing based on triple cation perovskite of Cs_0.0_(FA_0.83_ MA_0.17_)_0.95_Pb(I_0.83_ Br_0.17_)_3_ chemical deposition on vertically SnS_2_ nanosheets has been considered; this engineered photodetector exhibits significant performance in the wide spectrum range as well as fast photoresponse. The stability of the device has been investigated at 100% humidity and room temperature conditions, through a water resistance poly (methyl methacrylate) (PMMA) protective layer.

## Result and discussion

### Physical and chemical characterizations

**Figure 1:**
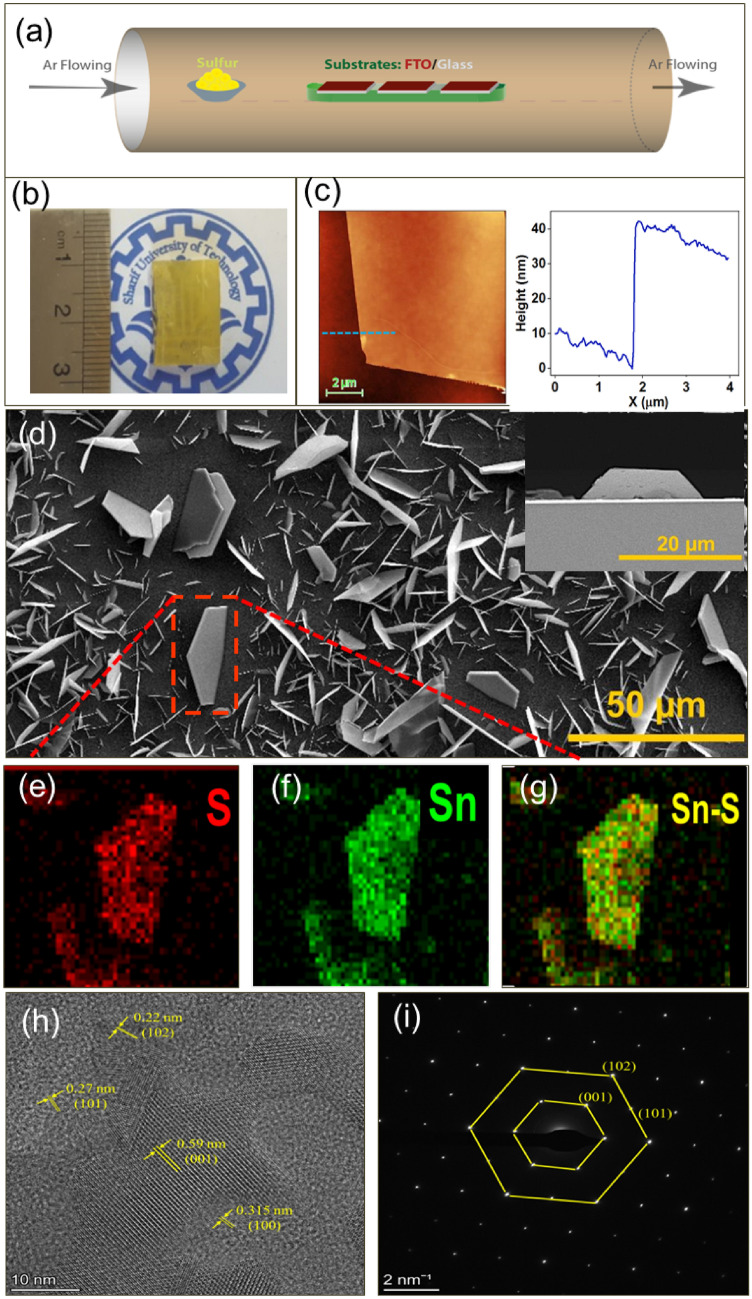
SnS_2_ nanosheet growth structural charactrizations. (**a**) Schematic of modified CVD using the FTO/glass as both the substrate and the Sn source for growing vertically SnS_2_ nanosheets. (**b**) Photograph of the sample of SnS_2_ nanosheets on the large scale FTO/glass substrate. (**c**) AFM images of SnS_2_ nanosheet on SiO_2_ substrate and the corresponding height profile. (**d**) FESEM analysis of the typical SnS_2_ nanosheets grown by CVD introduced by using 500 mg of sulfur source; the inset shows the l cross-section of 2D vertically SnS_2_ nanosheets on the FTO/glass. Elemental mapping image of (**e**) S, (**f**) Sn and (**g**) Sn-S atoms for typically grown nanosheet on FTO/glass substrate (**h**) The HRTEM and (**i**) selected area electron diffraction (SAED) pattern of a part of SnS_2_ nanosheets.

Here, we show that the vertical growth orientation of hexagonal with 2D SnS_2_ nanosheets through a standard CVD system at low temperature (500 °C) on FTO glass. Although, some reports shows vertically SnS_2_ nanosheets grown on the substrate at low temperature, here the FTO plays both as the substrate and tin source for SnS_2_ growth in our CVD setup (Fig. 1a). The proposed method is capable of producing SnS_2_ nanosheets in a large scale as shown in Fig. 1b. The FESEM analysis of the prepared samples depicts the vertically orientated morphology with clear semi-hexagonal shape including three or five crystal edges. The Fig. 1c shows a thickness of about 40 nm from an isolated SnS_2_ flake on Si/SiO_2_ substrate. The Fig. 1d illustrates the vertically SnS_2_ nanosheets on FTO as well as the thickness of about 5 µm, by applying 500 mg of the Sn source. Figure 1e-g demonstrate elemental mapping images of S, Sn and Sn:S atoms, respectively, using energy dispersive X-ray spectroscopy (EDS). The related homogenous elemental distribution reveals the proportional elemental ratio for S to Sn in the nanosheets (Fig. S1). Further analysis has been performed for as-grown SnS_2_ nanosheets using HRTEM (Fig. 1h). The interplanar lattice spacing can be extracted as 3.15 Å corresponding to (100) and (010) planes and the lattice spacing of 5.9 Å is corresponding to the basal plane of (001).

The selected area electron diffraction (SAED) pattern of the sample clarifies a single set of the perfect hexagonal pattern, indicating the crystal nature of the SnS_2_ nanosheet with the hexagonal crystal structure (Fig. 1i). The crystal planes in SAED are (100) and (010) planes with the angles of ~ 120° degree; the main exposed surfaces of SnS_2_ nanosheets are (001) basal planes. These results indicate good agreement with the previously reported lattice parameters of SnS_2_ crystals grown by CVD methods^15,31,32^.

**Figure 2:**
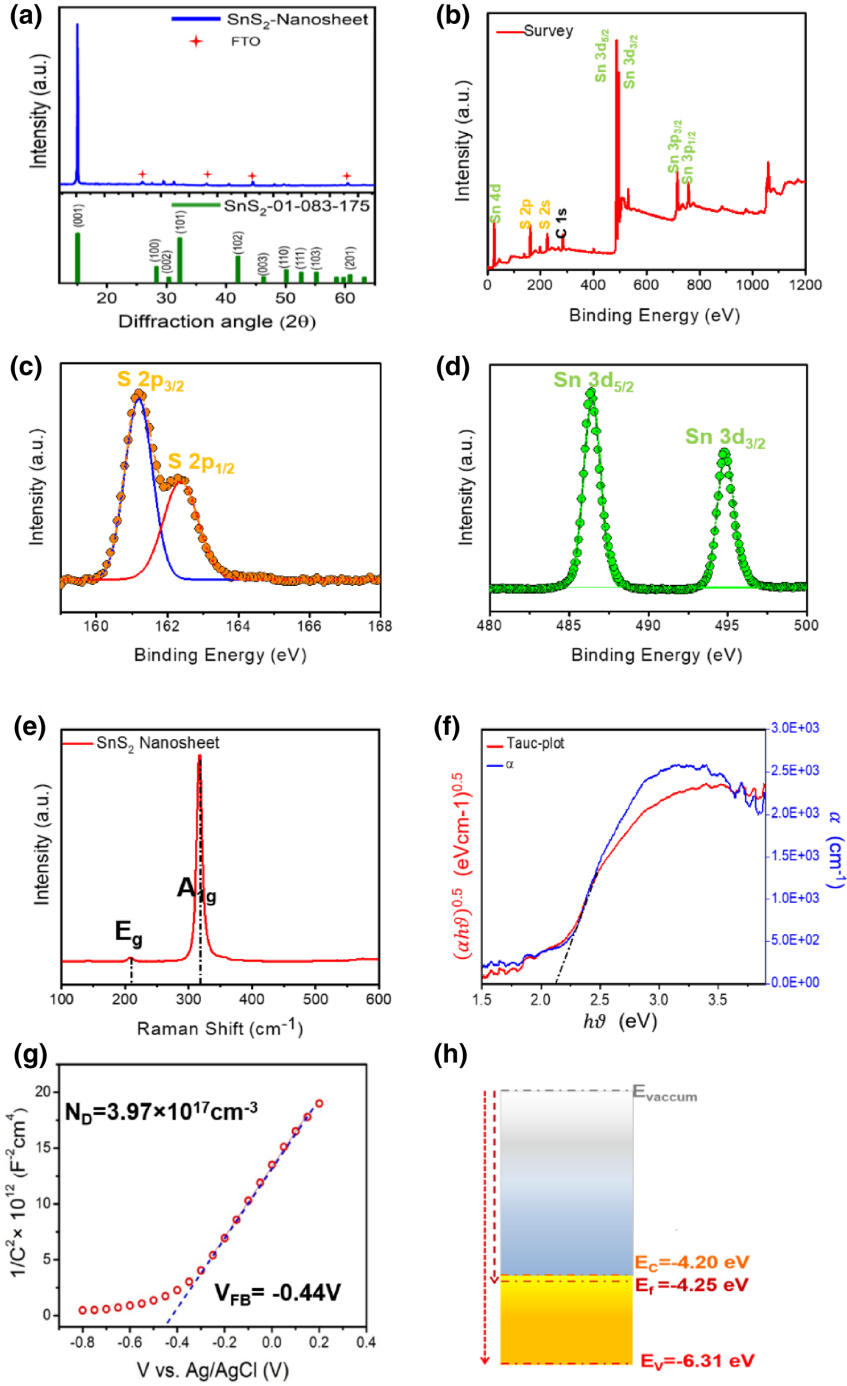
Crystal structure and optical properties of SnS_2_ nanosheet. (**a**) XRD pattern. (**b**) Full XPS spectra, (**c**) S 2p and (**d**) Sn 3d peaks of grown vertically SnS_2_ nanosheet on FTO, (**e**) The Raman spectra (**f**) Absorption evolution versus energy and Tauc plot of vertically grown SnS_2_ nanosheet on the FTO. (**g**) The Mott–Schottky analysis of vertically SnS_2_ nanosheets grown on FTO substrate in the 1 M aqueous Na_2_SO_4_ at f = 1 kHz, (**h**) The calculated energy levels of SnS_2_ nanosheet extracted by Mott-Schottky analysis.

The crystallographic structure of SnS_2_ nanosheets extracted from the diffraction pattern of XRD spectra, well-matched with a hexagonal structure of 2H-SnS_2_ nanosheets (JCPDS no 01-83-1705). Figure 2a shows diffraction peaks corresponding to (001), (002), (101), (102), (003), (110), (111) and (110) facets. It’s clear that (001) is the preferential orientation of crystal growth, which agrees with SAED pattern of SnS_2_ nanosheets. Moreover, based on Bragg’s equation, average spacing in the basal plane of SnS_2_ is about 5.8 Å which is consistent with the HRTEM analysis and disclose the (001) direction as the main basal planes of grown SnS_2_ nanosheets with lattice spacing distance of 5.9 Å. XPS analysis of the prepared SnS_2_ sample indicates the presence of the Sn and S elements, without any other additives (Fig. 2b). The high-resolution S 2p core level analysis at the binding energy of 161.2 eV and 162.3 eV shown in Fig. 2c, attributes to S 2p_3/2_ and S 2p_1/2_, respectively. The strong peaks around 486.4 eV and 494.8 eV (Fig. 2d) assign to Sn 3d_5/2_ and Sn 3d_3/2_, respectively is corresponding to chemical states of Sn^4+^ and S^2−^ of the SnS_2_^33^.

Raman analysis (Fig. 2e) displays two peaks located at 320 cm^−1^ and 208 cm^−1^ appointed to A_1g_ (out of the plane) and E_g_ (in-plane) modes of the SnS_2_, presenting an explicit fingerprinting of 2H phase^34^ of the SnS_2_ crystals in this work. These results confirm the growth of pure SnS_2_ nanosheets with high crystal and chemical quality. The diffusion transmission and reflectance spectroscopy of SnS_2_ on FTO/glass substrate was utilized to calculate the absorption coefficient spectra, using the following equation^35^

1 $$\alpha = \frac{{ - \ln \left( {\frac{T}{{1 - R}}} \right)}}{d}$$

2 $$\alpha h\nu = A(h\nu - E_{g} )^{{{\raise0.7ex\hbox{$n$} \!\mathord{\left/ {\vphantom {n 2}}\right.\kern-\nulldelimiterspace} \!\lower0.7ex\hbox{$2$}}}}$$

where $\alpha$, T , R, d, h, $\nu$, $A$ and E_g_ are absorption coefficient, transmission and reflectance, film thickness, Planck's constant, frequency of light and bandgap respectively. In Eq. (2), n = 4 represents an indirect bandgap semiconductor such as the SnS_2_ bulk sample^36^. As shown in Fig. 2f, the estimated optical band gap for the film containing SnS_2_ nanosheet arrays-grown by applying 500 mg of the tin source was obtained as 2.11 eV by extrapolating of the linear part of the ($\alpha h\nu$)^1/2^ versus photon energy plot. Moreover, Mott–Schottky (MS) analysis was utilized to calculate the donor density, as well as the conduction and valance band positions of the SnS_2_ nanosheets, relative to the vacuum energy level. The Donor density (*N*_*D*_) and flat band potential (*V*_*fb*_) can be estimated by MS equation^37^.

3 $$C_{Sc}^{ - 2} = \frac{2}{{N_{D} \varepsilon_{0} \varepsilon_{r} e_{0} }}\left( {V - V_{fb} - \frac{KT}{{e_{0} }}} \right)$$

where C_sc_ is the capacitance of the space charge layer, *e*_*0*_ is the electron charge; ɛ_r_ = 17.7 is the dielectric constant of the SnS_2_^38^, ɛ_0_ is the vacuum permittivity, V is the applied potential versus Ag/AgCl, *T* is the absolute temperature, and *K* is the Boltzmann constant. The positive slope of capacitance variation as the function of applied voltage (Fig. 2g), indicates the fabricated SnS_2_ nanosheet is an n-type semiconductor. The flat band potentials obtained by linear extrapolation of the MS plot is about − 0.44 V versus Ag/AgCl which stands for E_f_ as − 4.25 eV versus vacuum level energy. Carrier density of the SnS_2_ nanosheets is about 3.97 × 10^17^ cm^−3^; the conduction band energy of the n-type SnS_2_ versus absolute vacuum energy state (AVS) can be extracted through the following equation^38^

4 $$E_{CB} = V_{fb} + kT\ln \frac{{N_{D} }}{{N_{CB} }}$$

The N_CB_ is the effective density of the states close to the conduction band which can be evaluated as 7.32 × 10^18^ cm^−3^ within the electron mass of 0.43m_0_^39^. Using Eq. (4), for a bandgap value of 2.11 eV, the conduction and the valance band of the growth SnS_2_ nanosheets are positioned at 4.20 eV and 6.31 eV, respectively versus absolute vacuum standard level (Fig. 2h). These results are also in good agreement with other reports^40,41^.

### Crystal growth mechanisms

**Figure 3:**
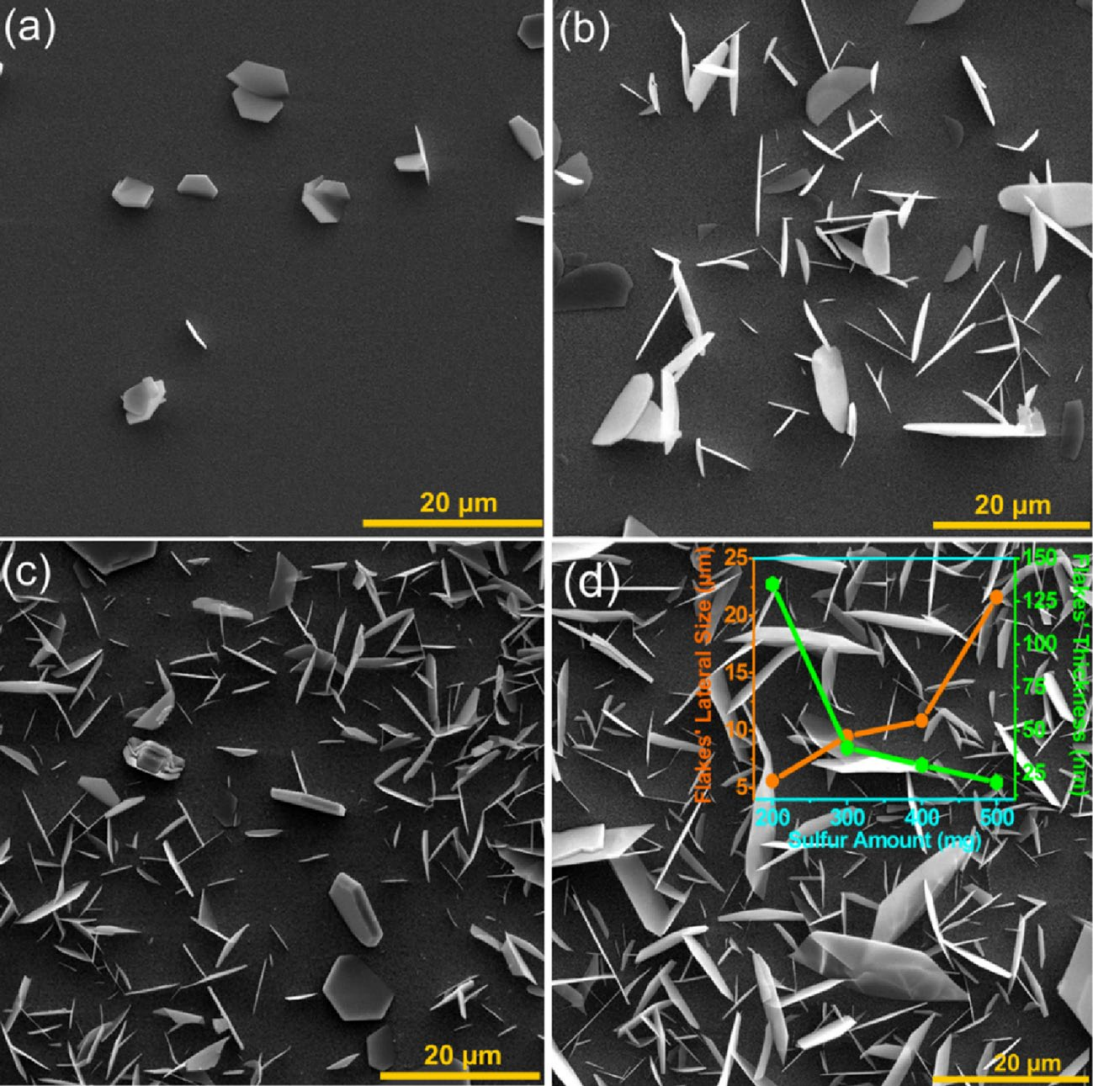
The effect of sulfure amount on the SnS_2_ nanosheets morphology on FTO substrate through modified CVD. (**a**) 200 mg, (**b**) 300 mg, (**c**) 400 mg and (**d**) 500 mg.The inset shows the evolution of average lateral size and thickness of grown SnS_2_ nanosheets by different sulfur amounts.

The influence of inter-distances between substrates and sulfur source position^42^, temperature and time parameters^40^ on the vertical growth of the SnS_2_ nanosheets in CVD method has been reported. Here, through a modified CVD process and using the FTO/glass as the substrate with no extra Sn source, the effect of sulfur precursor on the SnS_2_ nanosheet growth has been surveyed by FESEM analysis. In the case of S = 200 mg, as shown in Fig. 3a, a quite low density of SnS_2_ nanosheets have been distributed on FTO substrate; the average lateral size and thickness of the SnS_2_ with semi hexagonal structure was about, 5.6 µm and 135 nm, respectively (Figs. S2a and S3a). By increasing the sulfur source to 300 mg, the quantity of the SnS_2_ nanoflakes on FTO has been incredibly increased (Fig. 3b); the average lateral size of SnS_2_ nanoflakes increased to 9.5 µm while the thickness sharply decreased to 40 nm (Figs. S2b and S3b). However, by using further sulfur to 400 mg, the growth results in fairly full coverage of the FTO substrate (Fig. 3c) with no change in the average lateral size of the flakes while the edge thickness was thinned to 30 nm (Figs. S2c and S3c). Eventually, using 500 mg of the sulfur element led to full coverage of the semi-hexagonal SnS_2_ nanosheets (Fig. 3d); the average lateral size broadened to ~ 21.6 µm and the thickness was reduced to ~ 20 nm (Figs. S2d and S3d).

Therefore, the increment of the sulfur precursor leads to more SnS_2_ nanosheets with larger lateral sizes and sharper edges. A graph as an inset of Fig. 5d with 2Y axis summarizes these trends. The nanosheets showed relatively similar aspect ratios of semi hexagonal shapes due to high oriented growth in almost [100] and [010] rather than [001] direction^43^.

**Figure 4:**
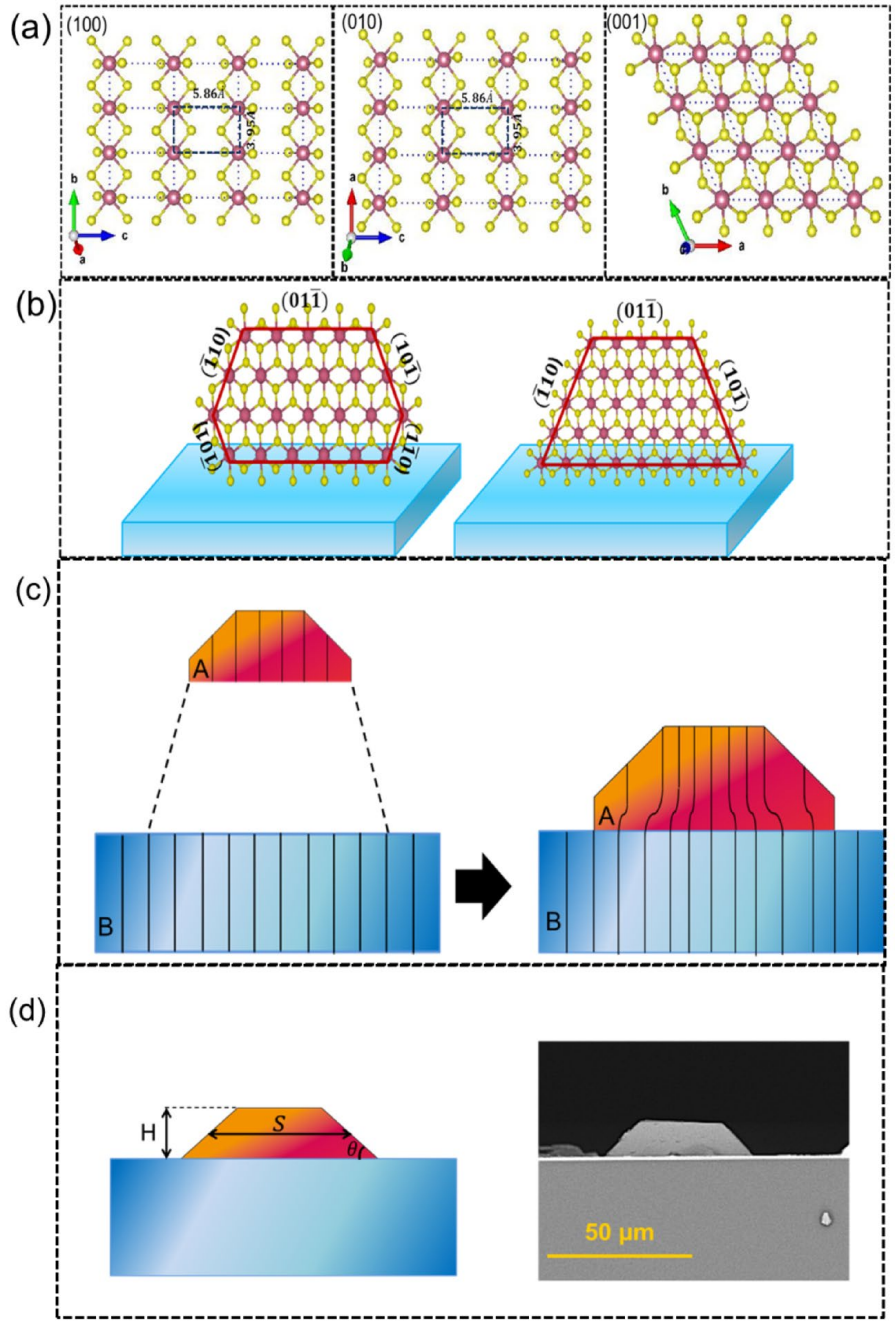
SnS_2_ crystal growth mechanism. Schematic of (**a**) Crystal structure model in different (hkl), (**b**) Formation of grown vertically SnS_2_ nanosheets with five and three crystal edges on the substrate (**c**) Pseudomorphic or coherently strained growth, to match the lattice constant of film (A) and substrate (B)^44^. (**d**) Schematic of grown polyhedral on the substrate with its parameters and FESEM analysis of the vertically grown SnS_2_ nanosheet on FTO.

Crystal growth of hexagonal SnS_2_ nanosheets can be discussed from two aspects: Bravais rule and surface energy theory. According to the Bravais rule, the most morphologically favourable crystal forms would be those planes having the highest reticular densities^44^. Based on XRD and HRTEM characterizations, SnS_2_ has a hexagonal layered structure with a unit cell of $a = b$ = 3.65 Å and $c$ = 5.9 Å. Similar to other TMDs structure, the Sn and S atoms are covalently bonded together in the layers, while the adjacent layers with the highest lattice distance of $c$, are stacked through weak Wan der Waals forces^45^ . The anisotropic nature of atomic bonding in SnS_2_ (Fig. 4a) leads to preferential growth velocity along [001], [010] and [100] directions. Since the lattice space of (001) is 5.9 Å, which is larger than the {010} planes, the highest reticular densities are related to {100}.

On the other hands, regarding surface energy theory, the growth of a crystal occurs through the plane which has the lowest surface energy based on the Gibbs principle in thermodynamic^46^. Hence, for the stable crystal nuclei with the constant free energy per unit volume, the surface energy should be minimized in a crystal that is bounded by *n* faces as in the following equation

5 $$\sum\limits_{i}^{n} {a_{i} g{}_{i} = \min }$$

where a_i_ is the area of the ${i}_{th}$ the face of the crystal and g_i_ is the surface energy per unit area of the i_th_ face. As the {001} plane of SnS_2_ may own the lowest surface energy (g_100_ = 0.034 eV/Å^2^ and g_001_ = 0.0065 eV/Å^2^)^47^, the growth of crystal should occur in (001) plane. As illustrated in Fig. 4b, after forming the SnS_2_ nucleus on the FTO, the new species favourably consolidate themselves into crystal lattice along the {100} planes rather than {001} planes due to much higher surface energy along [100] and [010] than the [001]^42,47^. Therefore, rapid transverse growth and slow vertical growth makes the (001) plane as the most exposed basal plane.

The lattice mismatch of nucleation A on the substrate affected the growth of the final structure (Fig. 4c). When the substrate and the film keep their different bulk lattice constants, the growth is stress-free, or incoherent. The growth is pseudomorphic or coherently strained when the film is stretched or compressed so that the film and substrate of the in-plane lattice constant are matched^48^. In the latter, the elastic energy of mismatch is released by the formation of islands or dislocations, partially or totally. Moreover, in this study, as the growth process has been performed at a relatively low temperature (500 °C), overcoming the energy barrier by SnS_2_ species is almost impossible and the lateral diffusion on the surface of the substrate is practically dismissed^42^. So, it seems that the stored elastic energy is released by the formation of 3D islands or misfit-dislocations. In the 3D islands mode, the Gibbs free energy is $\Delta g_{3D}$ which is related to the formation n density of 3D island. By regarding the square-based pyramidal island on the wetted substrates which is similar for the typical SnS_2_ nanosheet growth in this study (Fig. 4d), this parameter is equal to the following equation^44^

6 $$\Delta g_{3D} = n4SHg_{i} \csc \theta$$

where *H* and *S* are height and average lateral size of an island *A* on the substrate *B*; $\theta$ is the intersection angle between pyramid edge and substrate, and *g*_i_ is the surface energy of lateral face *i* of the polyhedral island. By adding the sulfur amount, the probability of the reaction and formation of nuclease increases, so the surface energy consumed per unite area increases. Based on the above equation, by increasing the $\Delta g_{3D}$ in the same height of nuclease, it is expected that the lateral size of pyramided nuclease increases. We observed this phenomenon for the SnS_2_ nanosheets grown on the FTO substrate. Subsequently, based on these equations, it is predictable that the thickness of the SnS_2_ nanosheet decreases by adding the sulfur amount through the introduced CVD method.

As the last point, the Wulff’s theory could be taken into account to understand the morphology of SnS_2_ nanosheet in this research. In this regard, the equilibrium shape of free crystal *A* can be described by introducing the constant ratio of $\lambda = \frac{{g_{j} }}{{h_{j} }}$ between the surface energy *g*_*j*_ of each $j$ facet bounding to the crystal of the given volume and distance *h*_*j*_ of that facet from a common point inside the crystal so-called Wulff’s point^44,46^.

It follows that for a free crystal growing near the equilibrium condition, the crystal shape for different λ (different sizes) is self-similar around the Wulf's point (Fig. S4a). Hence, the constancy of the shape ratio can be expressed as

7 $$r_{i} = \frac{{h_{A} }}{{h_{i} }} = \frac{{g_{A} }}{{g_{i} }}$$

For a supported crystal *“A”* on substrate *“B”*, the equilibrium shapes were determined by minimization of surface and interface energies. In the absence of misfit between A and B, the self-similarity around the common point S on the substrate has been sustained (Fig. S4b) which is a result of the size independence of shapes ratio (Wulff-Kaishev theorem)^44,46^.

8 $$r_{i} = \frac{H}{{h_{i} }} = \frac{{2g_{A} - \beta }}{{g_{i} }}$$

where $H = h_{A} + h_{AB}$ is the emerging height of crystal; *h*_*AB*_ is the common distance of the interface surface from all the pyramid apexes (Wulff’s point) taken positive or negative according to whether the Wulff’s point is outside or inside the substrate, respectively; $\beta_{AB}$ is the specific adhesion free energy of face $\mathrm{i}=1$ of *A* on *B* and $g_{A}$ is the corresponding free energy of this face.

For the case of a strained polyhedral crystal, A supported on substrate *B*, similar to SnS_2_ nanosheet growth in this study, elastic bulk energy plays an important role. Assuming coherence growth, the elastic strain accumulates both in crystal and in the nearby region of the substrate during growth (Fig. S4c), which is reduced by relaxation-energy factor *R*. In this case, by minimization of the free energy at the constant volume, the equilibrium shape of the crystal can be gained, which contains the elastic energy. The shape ratios for crystal *“A”* with volume *V*_*A*_ having free facets of area *S*_*i*_ and a contact area *S*_*AB*_ with substrate “B” of area *S*_*B*_, based on generalized Wulf-Kaishew theorem, obeys the following equation^44^

9 $$r_{i} = \frac{H}{{h_{i} - h_{A} \cos \theta_{i} }} = \frac{{2g_{A} - \beta + C_{1} V_{A} \frac{\partial R}{{\partial S_{{AB_{{}} }} }}}}{{g_{i} - g_{A} \cos \theta_{i} + C_{2} V_{A} \frac{\partial R}{{\partial S_{i} }}}}$$

where *C*_*1*_ and *C*_*2*_ are the constant elements. Thus, the shape ration depends on the size of the crystal and there is no more self-similarity of equilibrium shape, consequently.

In our catalyst-free growth, as no kinks exist on the substrate, the nucleation can occur at any site on it^43^. The strained polyhedral SnS_2_ nucleus crystal deposited on lattice mismatches of FTO substrate and asymmetric SnS_2_ nanosheets growth happened to release its stored elastic energy. These processes occur for nucleation-growth as layer by layer for SnS_2_ flakes^15,42,43,47^.

The vertically grown crystal is strongly suitable for planar electronic devices because it provides a higher density of SnS_2_ nanoflakes with enormously exposed surface/edges per unit area^12^. Due to this fact, we have used this fascinating structure as a primary material for photodetectors.

### Photodetector operation

#### Pristine SnS_2_ based photodetector

**Figure 5:**
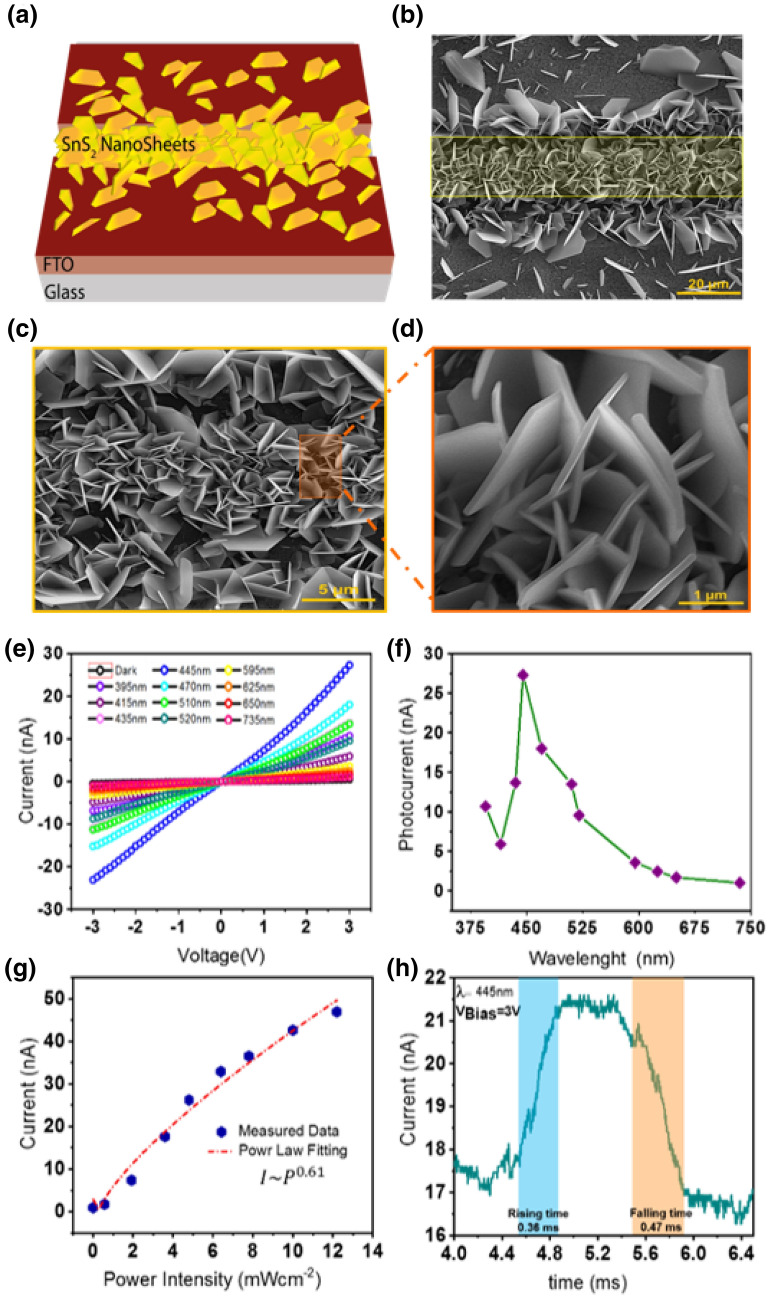
Pristin SnS_2_ nanosheet based photodetector. (**a**) Schematic and (**b**), (**c**), (**d**) Different magnitude of FESEM analysis of grown vertically gown SnS_2_ nanosheets on the laser patterned FTO through CVD process. (**e**) The evolution of current versus applied voltage (**f**) The photocurrent at 3 V bias, for different wavelength at the light intensity of 5 mW/cm^2^ (**g**) Dependence of photocurrent with different light intensity of λ = 445 nm at 3 V, regarding power law fitting and (**h**) Time-resolved photoresponse at 5 mW/cm^2^ light intensity of λ = 445 nm and at 3 V, for planar SnS_2_ photodetector.

As vertically species provides enormously exposed surface/edges per unit area^12^, we have used this fascinating SnS_2_ structure as a primary material for low-cost planar photodetector. This photodetector been developed by using the FTO/glass with 25 µm etched strip, in which two sides of the etched area act as the planar electrical contacts thanks to the highly conductive properties of FTO film. In this study, by using the introduced CVD method, grown SnS_2_ nanosheets helps to bridge between two separated parts on the substrate, as shown in Fig. 5a,b. So, some traditional steps such as TMD’s flake transferring, lithography, oxygen plasma process, and metallization process for electrical contacts such as Cr/Au or Pt deposition, have been avoided which offer photodetector fabrication with no need for high vacuum condition process. The higher magnification of the vertically SnS_2_ nanosheets has been shown in Fig. 5c. The interconnection of these structure is more clear in the Fig. 5d.

The schematic of the planar photodetector is illustrated in Fig. S5a, where the light is illuminated from the backside. Figures 5e and S5b present the current–voltage characteristics of the SnS_2_ based photodetector in darkness and under illumination. The photocurrent is indicating Schottky like junction at FTO-SnS_2_ contacts, due to junction of FTO with a higher work function (− 4.4 eV) in contact with the SnS_2_ semiconductor layer with lower conduction energy level (− 4.2)^15^ as shown in inset of Fig. S5b.

The current evolution vs. different light wavelengths at 3 V (Fig. 5f) presents the highest photocurrent about 27.3 × 10^–8^ A at 445 nm of illumination. Figure 5g, displays the dependence of photocurrent on the light intensity at 445 nm wavelengths, which can be described by power-law as $I\sim {P}^{\beta }$ , where $P$ is the light intensity and $\beta$ is the parameter related to the trap states on the photovoltaic materials. The trap states originating from sulfur vacancies and/or charged impurities in the SnS_2_ .More trap sites could be filled with photogenerated charge carriers as the light intensity rises^49^. By fitting the measured data by the power-law equation, the value of $\beta$ is 0.61 which is close to the other reports^15,49^.

Next, the photoresponse time as one of the critical parameters of photodetector was tested at wavelengths of 445 nm with 5mW/cm^2^ light intensity at 3 V applying bias. Figure S5c,d display the time-resolved photoresponse curve under illumination/dark exposure cycles for a long time at 1 Hz and 1 kHz, respectively. It is obvious that the photodetector has reasonable stability and steady response toward the incident light. The one cycle of current vs. time of SnS_2_ based photodetector at 1 kHz (Fig. 5h), describes a fast rise time ${(\tau }_{r}$) about 0.36 ms and decay time (${\tau }_{d}$) of 0.47 ms. This record of the rise and decay times that are both above tenth of milliseconds reflects an enhancement by at least two orders of magnitude relative to other parallel and vertical SnS_2_ nanosheet based photodetectors^34,49^.

#### SnS_2_/TCLP photodetector

**Figure 6:**
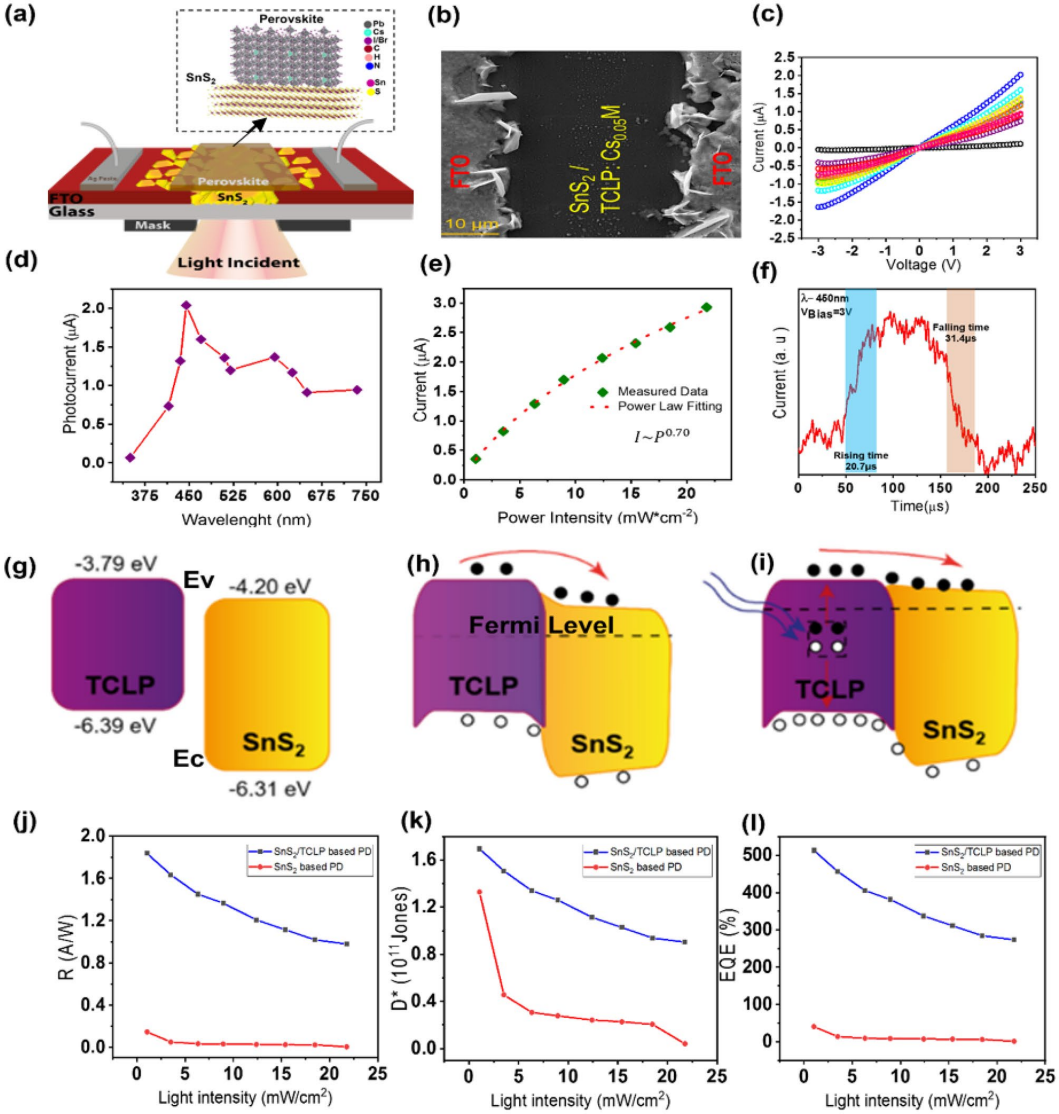
Photoresponces of SnS_2_/TCLP device. (**a**) The schematic of the planar SnS_2_/TCLP photodetector. (Inset: The SnS_2_/TCLP layer in its atomic structure). (**b**) The top view FESEM of deposited TCLP layer on grown SnS_2_ nanosheet on patterned FTO (**c**) The I–V curves and (**d**) photocurrent at 3 V bias, for different wavelengths at the light intensity of 5 mW/cm^2^ (**e**) Dependence of photocurrent with different light intensities of λ = 445 nm at 3 V, regarding power-law fitting; and (**f**) Time-resolved photoresponse at 5 mW/cm^2^ light intensity of λ = 445 nm at 2 kHz on/off cycle. The band diagram energy of the SnS_2_ and TCLP layers (**g**) before connection together, after connection together (**h**) in the dark (**i**) under the illumination. The evolution of photodetector parameters, (**j**) responsivity, (**k**) EQE% and (**l**) detectivity as a function of light intensity for Pristine SnS_2_ and SnS_2_/TCLP based photodetectors at 445 nm of incident light with different intensities.

The Cs_0.05_(FA_0.83_MA_0.17_)_0.95_Pb(I_0.83_Br_0.17_)_3_, abbreviated as Cs_0.05_M, where M stands for mixed perovskite-with appreciated electrical properties and remarkable stability besides its high light absorption coefficient, is a promising candidate for optoelectronic devices^25^. The XRD analysis of this material (Fig. S6a) confirms that crystalline planes of Cs_0.05_M perovskite layer have been formed properly. The estimated optical bandgap is about 1.63 eV which is in agreement with the clear peak of photoluminescence spectra in 765 nm (Fig. S6b). Therefore, the Cs_0.05_M should be a potential candidate to be utilized as an absorbing layer with SnS_2_ nanosheets due to the well-matching of energy bandgaps^25^. In this research, facile, low cost and large scalable planar design of SnS_2_/TCLP photodetector has been fabricated through deposition of the TCLP on the SnS_2_ nanosheets. The schematic of this photodetector is shown in Fig. 6a. The inset Fig. 6a indicates the SnS_2_/TCLP layer in its atomic structure. The top-view FESEM analysis of SnS_2_/TCLP (Fig. 6b) displays full embedding of the SnS_2_ nanosheets grown on patterned FTO/glass by TCLP film. While the thickness of pure SnS_2_ (5 µm) layer and SnS_2_/TCLP (5.5 µm) (Fig. S7a) layers are almost the same, the SnS_2_/TCLP indicates more light absorption in the range of 320–740 nm, in comparison with pristine SnS_2_ and perovskite layers (Fig. S7b). It can be expected that the SnS_2_/TCPL layers presents enhancement in the photocurrent rather than pure SnS_2_ and TCLP films.

Figures 6c and S7c show the current evolution of the photodetector under the illumination of different wavelengths under the same intensity of 5mW/cm^2^. As Fig. 6d displays by applying the 3 V and variation the irradiation wavelength from 395 to 450 nm, the photocurrent raised and reached a maximum value at 445 nm. By increasing the wavelengths of illumination up to 520 nm, the current slightly decreases and at 595 nm, the current falling takes place for longer wavelengths. A gentle decrease of the photocurrent by wavelength variation for the device can be attributed to the characteristics of light absorption of the perovskite layer in the visible region. The current variation vs. light intensity evolution fitted by the power-law equation gives $\beta$ = 0.7 (Fig. 6e). The increment of this parameter compared to that of pristine SnS_2_ based device indicates the lower carrier recombination process in SnS_2_/TCLP device. The photocurrent evolution versus time with on/off modes of 1 Hz (Fig. S7d) and 2 kHz (Fig. S7e) frequency under the wavelength of 445 nm at 3 V bias represent repeatable and stable performance with the on/off ratio of ~ 40. One sequence of 2 kHz on/off cycle on SnS_2_/TCLP device under light illumination of 445 nm, as shown in Fig. 6f, indicates the rise and falling times shortened one order of magnitude, as fast as 20.7 µs and 31.4 µs, respectively.

Increasing the current in darkness or under light irradiation is due to enhancement of the charge carriers’ concentration by the evolution of the band structure of the SnS_2_ nanosheets in junction with the TCLP layer. To investigate the effect of the TCLP layer on the photodetection performance of the SnS_2_/TCLP photodetectors, a pristine TCLP film based photodetector is also prepared (Fig. S7f.). By comparing the Fig. S7f. and S7c, the dark current for both devices estimated in the same ranges with the order of a few tens of the nA, while the photocurrent enhancement for SnS_2_/TCLP and TCLP enhanced to the 1.64 µA and 0.82 µA under the illumination of 445 nm, respectively. This highlights the role of SnS_2_ nanosheets. To further realize the mechanism of improved photoresponces in hybrid SnS_2_/TCLP device, the energy band alignment of SnS_2_ and TCLP in the dark and under illumination should be considered. The energy levels of the SnS_2_ nanosheet estimated based on Mott–Schottky analysis in junction with TCLP is shown in Fig. 6g before contact together. After the physical connection of these two layers, the interface of the SnS_2_/TCLP, band bending occurs with band offset at the valence and conduction bands (Fig. 6h) which facilities charge transferring in one preferred direction. Different Fermi levels of these two materials also lead to the formation of depletion regions and provide a built-in electric field to drive the separated electron–hole pairs in the TCLP film. When the light is illuminated, electron–holes pairs are created in the TCLP layer (Fig. 6i). Due to the existence of the built-in electric field, electrons are transferred to the SnS_2_ nanosheets, while holes are restricted in the perovskite layer. The accumulated charge carriers in the SnS_2_ nanosheets increase (decrease) the Fermi level in the SnS_2_ (TCLP), respectively^50^. This engineered band bending results in more photo-charges creation/transferring at the interface toward metallic contacts.

The photocurrent generated per unit power of incident light on the effective area-photoresponsivity (R)-sensitivity of the photodetector-specific detectivity (D*)- and the number of electrons detected per incident photon-external quantum efficiency (EQE)-are the key parameters of photodetectors which calculate through following equations:

10 $$R = \frac{{I_{ph} }}{{P{}_{{_{0} }}}}$$

11 $$D^{*} = \frac{{RS^{{{\raise0.7ex\hbox{$1$} \!\mathord{\left/ {\vphantom {1 2}}\right.\kern-\nulldelimiterspace} \!\lower0.7ex\hbox{$2$}}}} }}{{(2eI_{d} )^{{{\raise0.7ex\hbox{$1$} \!\mathord{\left/ {\vphantom {1 2}}\right.\kern-\nulldelimiterspace} \!\lower0.7ex\hbox{$2$}}}} }}$$

12 $$EQE = \frac{{hcR_{\lambda } }}{e\lambda }$$

where *I*_*ph*_ is *I*_*p*_* (light current)*-*I*_*d*_* (dark current)*, *P*_*0*_ is the light intensity; *S* is the effective illuminated area which here is about 13.5 × 10^–9^ m^2^; $e$ stands out the elementary charge; $h$ is Plank’s constant; $c$ represents the light velocity; $R$ is the responsivity; and λ is the incident light wavelength^49,50^. Figure 6j, h and l demonstrate the evolution of the R (AW^−1^), EQE % and, D* (Jones) as the function of incident light intensity at 445 nm for the photodetectors based on pristine SnS_2_ and SnS_2_/TCLP. These graphs show maximum values of responsivity of 140 mAW^−1^, 41% as external quantum efficiency, and detectivity of 1.33 × 10^11^ Jones for pristine SnS_2_-based device, while these parameters enhanced to 1.84AW^−1^, 513% and 1.69 × 10^11^ Jones, respectively for SnS_2_/TCLP photodetectors. Moreover, the photodetector’s parameters for three SnS_2_, TCLP and SnS_2_/TCLP based photodetectors has been evaluated through different light wavelength illumination at 5 mW/cm^2^ (see Fig. S7g, h and i). These improvements are assigned to the enhancement of charge carrier production and better separation due to the well-engineered band alignment of SnS_2_ and three cationic perovskite.

Although the calculated R and D* parameters for SnS_2_/TCLP device is similar to hybrid structure of WS_2_ or MoS_2_ with perovskite films (Table 1), the proposed SnS_2_/TCLP fabricated using a simple and scalable approach, indicates very fast responses in comparison with the other TMD/perovskite devices.

**Table 1:** A summary review on some published reported parameters of TMDs/perovskite-based photodetectors.

Structure	R(A/W)	D*(Jones)	Rising/falling time	Fabrication method	Intensity @ wavelength @ applied voltage	Refs
CH_3_NH_3_PbI_3_:MoS_2_ nanohybrids	0.69	1.94 × 10^12^	50/16 ms	Chemical route synthesis and **mm** size device with photolithography& high vacuum deposition	0.051 (mW/cm^2^) @532 nm @3v	^51^
MoS_2_/CsPbBr_3_ nanohybrid	4.4	2.5 × 10^10^	0.72/1.01 ms	Mechanical transferring of the **µm** size flake with photolithography & high vacuum deposition	20 μW/cm^2^ @442 nm @10 V	^50^
WS_2_/CH_3_NH_2_PbI_3_	2.3	2.3 × 10^12^	2.7/7.5 ms	CVD synthesis the **µm** size flake with photolithography & high vacuum deposition	0.5 mW/cm2@480 @5 V	^27^
MoS_2_ QD/MAPbI_3_ film	–	5 × 10^11^	2.5 s	Epitaxial growth process of QD **mm** size with photolithography& high vacuum deposition	2.5 μW/cm2 @1200 nm @10 V gate	^52^
CH_3_NH_3_PbI_3_/MoS_2_ with planar design	1.6	–	356/204 ms	CVD synthesis the **µm** size flake with photolithography & high vacuum deposition	3.5 mW/cm^2^ @white light @ 1 V	^53^
SnS_2_/TCLP	1.8	1.69 × 10^11^	20 µs/31 µs	Laser patterned FTO and one step CVD synthesis of flakes in **mm** size	1.05 mW/cm^2^@ 445 nm @ −3 v	This work

#### Stability test

As the stability of the perovskite-based optoelectronic device in humidity has always been a challenge, at the next step, we tested the stability of the SnS_2_/TCLP encapsulated device. Although, the literature shows that conventional conducting polymer or small molecules as the protecting layer^54^, using the stable, eco-friendly and inexpensive material for protecting the perovskite layer through a cost-effective method, is still under debating^55^. In this study, the PMMA layer with low water absorptivity (0.3%)^56^, has been used as a protecting layer on the SnS_2_/TCLP device. The PMMA layer due to its interring cross-linked network can delay the degradation of the TCLP layer from oxygen and moisture of environmental conditions^55^. Since the light beam is illuminated from the backside of the photodetector, the protective layer should not be optically perturbed due to incident light.

**Figure 7:**
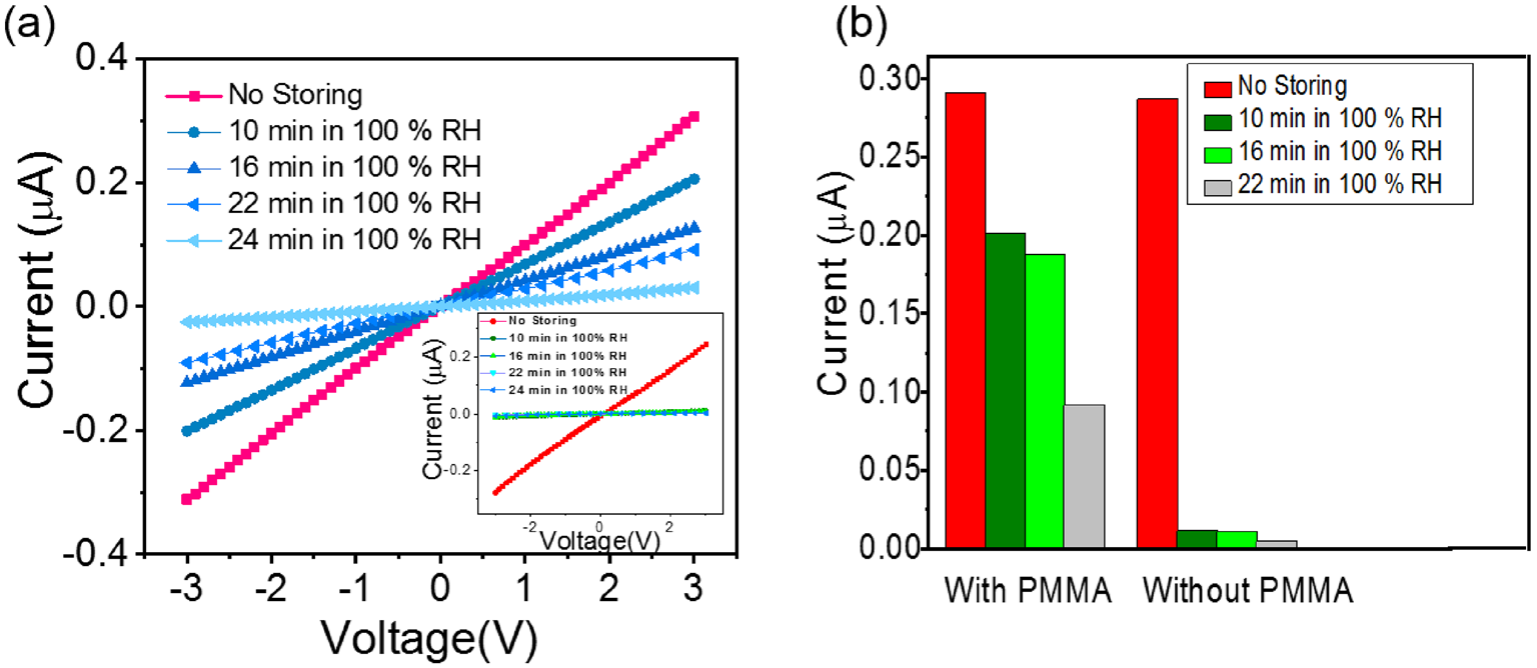
Stability test of the SnS_2_/TCLP photodetector. (**a**) I–V graph of planar SnS_2_/TCLP/PMMA photodetector, the inset graph shows the I–V result of unprotected SnS_2_/TCLP photodetector for different times storing from 0 to 24 min. (**b**) The trend of maximum current of protected and unprotected photodetectors with and without PMMA layer keeps for different times of 0, 10, 16, 22 min, under 100% humidity and room temperature; the light illumination is 445 nm with 5 mW/cm^2^.

The current–voltage graphs of the protected and unprotected photodetectors under the wavelength of 445 nm with 5 mW/cm^2^ were measured for different storing time in the 100% humidity and room temperature, as shown in Fig. 7a. The inset graph shows the *I–V* characteristics of the unprotected photodetector. The trend of current degradation percentage ($\frac{I}{{I}_{0}}*100$) at a bias voltage of 3 V, illustrates in Fig. 7b, which indicates that for 10, 16 and 22 min, the current degradation of the protected photodetector are 70%, 64.5% and 31.5% while these corresponding values decreased to 4%, 3.5 and 1.5%, respectively. Figure S8 shows a picture of photodetectors with and without PMMA layer, after storing in 100% humidity and room temperature condition for 10 min.

The clear change in the perovskite layer from dark brown to yellowish (Fig. S8) indicates the decomposition of Pb-I in the TCLP layer, as reported for the decomposition of MAPbI_3_^57^, which confirms the existence of moisture in the unprotected device. Therefore, the 20% PMMA, with low water absorptivity, is applicable as the protecting layer for photodetector or other optoelectronic devices through a simple solution method.

## Conclusion

In summary, the vertically SnS_2_ nanosheets have been grown directly through a low-temperature CVD process on FTO/glass with no extra Sn source. The mechanism study showed the growth of the polyhedral structure of the SnS_2_ crystals on the FTO that could be related to elastic energy and the mismatching between SnS_2_ nanosheets crystals and substrate. As the vertically TMDs have high densities of exposed surface area and edges per unit area, it should be beneficial for optoelectronic devices. In this research, the cost-effective photodetector based on vertically grown SnS_2_ nanosheets has been presented using laser scribed FTO substrate and CVD method. To enhance the device performance, in terms of light absorbance and carrier separation, three cationic perovskite layer of Cs_0.05_(FA_0.83_MA_0.17_)_0.95_Pb(I_0.83_Br_0.17_)_3_ with proper band alignment has been deposited on SnS_2_ nanosheets. the photodetector shows an impressive enhancement in parameters including high responsivity of 1.84 AW^−1^, EQE = 513% and detectivity = 1.69 × 10^11^ Jones under the incident light of 445 nm with 5 mW/cm^2^, with rising and falling time of ~ 20.7 µs and 31.4 µs, respectively. Besides, a protected (with PMMA layer) and unprotected SnS_2_/TCLP photodetectors have been investigated for different storing times at 100% humidity conditions in which the protected device showed much more stability than unprotected device.

## Experimental method

### Substrate preparation

The FTO/glass substrates (TCO30-10, 10 Ω/sq) were cleaned using a sequential cleaning process with detergent, acetone and ethanol in an ultrasonic bath for 10 min and 5 min sequentially, and finally rinsed by DI water and dried under Nitrogen stream.

### Growth of vertically aligned SnS_2_ hexagonal sheets

The cleaned and pattered FTO/glass substrates were sulfurized in a quartz tube furnace under argon atmosphere. The sulfur contents were varied to study the SnS_2_ nanosheets growth. In this case, elemental S powder was taken into a ceramic crucible for several amounts of 200, 300, 400 and 500 mg, in each separated experiments, while the substrate was kept 20 cm away from downstream.

Before sulfurization process, the trace of oxygen in the quartz tube was removed by purging the pure argon gas for 30 min; then the sample sulfurized at 500 °C for 60 min, eventually. The sulfur vapor specie (S) was incorporated by the tin source in the FTO substrate and then naturally was cooled to room temperature.

### Perovskite precursor solution

The organic compound, methylammonium bromide (MABr) and Foramidinium iodide (FAI), Cesium iodide (CsI) were purchased from Sigma Aldrich, and the other solvents were acquired by the Merck Company.

The perovskite precursor solution were prepared by (1.5 M) PbI_2_, (1.5 M) PbBr_2_, (0.5 M) FAI, and (0.5 M) MABrin anhydrous DMF:DMSO:(4:1). The CsI standard solution (1.5 M in DMSO), was stirred overnight at room temperature and then added into the precursor solution with an equal volume percentage. The final precursor solution of triple cation lead perovskite (TCLP), Cs_0.05_(FA_0. 83_ MA_0.17_)_0.95_Pb(I_0.83_ Br_0.17_)_3_ was stirred overnight. All the process has been done in ambient condition and at room temperature.

### Device fabrication

To fabricate the planar photodetector, engraving process of the FTO crystalline layer performed on FTO/glass substrate. In this regard, a ~ 25 µm gap on the FTO substrate created by physical etching using laser Nd:YAG laser, QCW fiber, wavelength of 1064 nm and 10 Watt laser cutter (Connect laser technology Co. LTD). The laser-engraved FTO/glass substrate was used in CVD process along with 500 mg of sulfur element 2 (Fig. S9a-c)as described in the “Experimental method” section. After CVD procedure, the SnS_2_ nanosheets growth on the substrate and filled the laser groove (Fig. S9d) To fabricate the SnS_2_/TCLP PD, the TCLP deposition was done through two-step spin-coating process at 1000 rpm and 4000 rpm for 10 s and 30 s, respectively, while the chlorobenzene anti-solvent was mildly casted at the final 5 s of the second step (Fig. S9e). The film was then baked at 100ºC for 1 h (Fig. S9f.). To make electrical contact, in all PDs (pure SnS_2_, pure TCLP and SnS_2_/TCLP,), the grown SnS_2_ nanosheets on FTO, were mechanically removed from both sides of the laser patterned gap. And then, to provide electrical contacts, the Ag paste was placed onto both side of the laser etched areas. Also, a mask with the effective area about 1.35E^−6^ cm^2^ has been regarded and finally, the incident light illuminated from backside of the fabricated PD as shown in Fig. S9g.

### Stability test

The prepared 20% wt PMMA in chlorobenzene as the protective layer was spin-coated on the perovskite/SnS_2_ device at a rotation speed of 6000 rpm for 90 s and then dried for 30 min at room temperature. The non-protected and protected photodetector were stored under 100% humidity at room temperature for 10 to 22 min. Next, the *I–V* measurement of both photodetectors was performed for the same storing time under bias voltage range of − 3 V to 3 V under illumination (wavelength of 445 nm at 5mW/cm^2^ by LED source).

### Characterization

The surface morphologies of the isolated SnS_2_ sheet were studied by atomic force microscopy (AFM) (Park Scientific CP-Research, VEECO) in tapping mode. Field emission scanning electron microscope (FESEM, TESCAN, MIRA3), energy dispersive X-ray analysis (Oxford instrument) and high-resolution transmission electron microscopy (HRTEM, JEOL, Model JEM-2010F UHR) were used to study morphology, elemental analysis and lattice distance of the SnS_2_ and SnS_2_/perovskite heterojunction, respectively. X-ray diffraction (X’ Pert Pro, PANalytical) was performed to study of crystallization and structural characteristics of samples. X-ray photoelectron spectroscopy (XPS) was utilized to investigate chemical states of the flakes while the data acquisition was made by using a hemispherical analyzer with an Al Ka X-ray source (hν = 1486.6 eV) operating at a vacuum better than 10^–7^ Pa. Optical properties were explored by diffuse reflectance/transmittance spectroscopy (Avantas- Avaspec-2048-TEC). Raman spectrum analysis was performed using a 532 nm laser (Explora, Horiba). The mott-Schottky analysis was carried out by a three-electrode setup with Ag/AgCl in 3 M saturated KCl and Pt wire as the reference and counter electrode, respectively and the electrolyte was an aqueous 1 M Na_2_SO_4_. The potential was swept in the range of stability diagram with an AC signal of 10 mV of amplitude superposed on the DC component using a Potentiostatic-galvanostatic system (Auto-lab system, PGSTAT30).

The opto-electrical/electrical measurements of the devices were carried out using KEITHLEY 6487 picoammeter voltage source instrument. Light sources were arranged as an array of several LEDs from an extended range of UV to Vis wavelengths (395–740 nm). To measure the rising and falling time of the photodetector, an operational amplifier circuit and an oscilloscope have been utilized. A pulsed light has been used controlled by GWINSTEK GDS-1052-U oscilloscope along with a current to voltage converter circuit (Fig. S10). The amount of the current in the dark and light has been calculated via the amount the out-put voltage and the resistance in the circuit.

## Supplementary Information

Supplementary Information.

## Data Availability

Derived data supporting the findings of this study are available from the corresponding author on request.
