# Supplementary material for: Ultrafast and stable planar photodetector based on SnS2 nanosheets/perovskite structure
Source: Sci Rep. 2021 Sep 29;11:19353. doi: 10.1038/s41598-021-98788-x (PMC8481477; doi:10.1038/s41598-021-98788-x)
Supplement: Supplementary file 1 — Supplementary Information. [file 41598_2021_98788_MOESM1_ESM.docx]

Supplementary Information:

**Ultrafast and Stable Planar Photodetector Based on SnS_2_ Nanosheets/Perovskite Structure**

Leyla Shooshtari^1^, Ali Esfandiar ^1^*, Yasin Orooji ^2^, Mahmoud Samadpour ^3^, Reza Rahighi ^4^

1. Department of Physics, Sharif University of Technology, Tehran P.O. Box11155-9161, Iran

2. College of Materials Science and Engineering, Nanjing Forestry University, Nanjing 210037, PR China

3. Department of Physics, K.N. Toosi University of Technology, Tehran, 15418-49611, Iran

4. SKKU Advanced Institute of Nano-Technology (SAINT), Sungkyunkwan University, 2066, Seobu-ro, Jangan-gu, Suwon, Gyeonggi-do 16419, Republic of Korea

* Email: e*sfandiar@physics.sharif.edu*

**Fig. S1. Elemental spectrum of the as grown SnS_2_ flakes:**

|  |
| --- |
| Fig. S1. EDX analysis of grown SnS_2_ nanosheets through modified CVD method. |

**Fig. S2 and Fig. S3. Effect of sulfur precursor:** Investigation the dependence of average lateral size of SnS_2_ nanosheets by using the different amount of sulfur source in the CVD method, in this research has been performed, using FESEM analysis. For calculating the average of lateral size of the SnS_2_ flakes, the Image J software has been used.

| 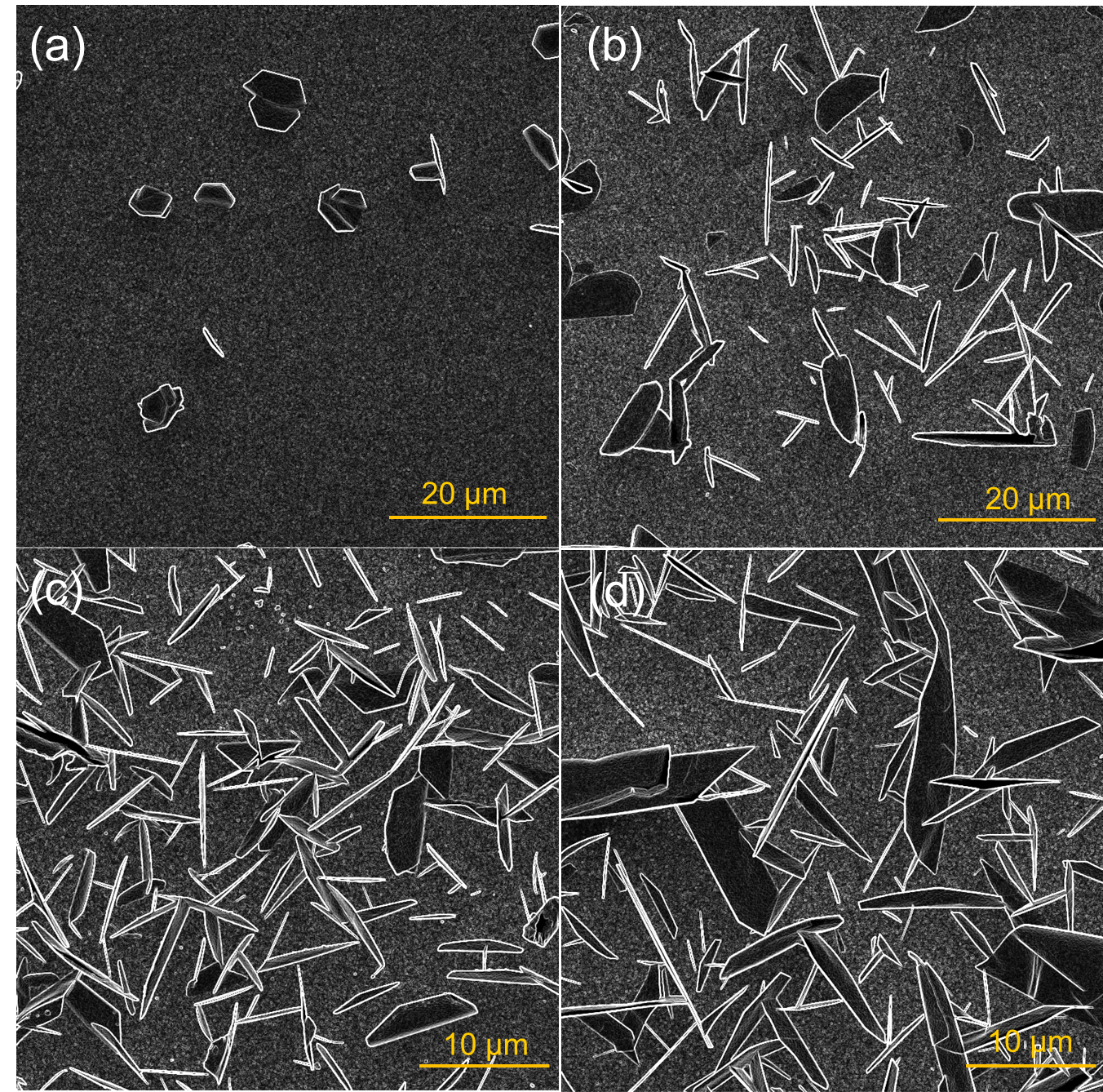 |
| --- |
| Fig. S2. The influence of sulfur amount on the lateral size of SnS_2_ nanosheets  FESEM analysis of vertically grown SnS_2_ nanosheets on FTO substrate through modified CVD method, by using different amount of sulfur of a)200mg, b)300mg, c)400mg and d)500mg. for calculating the average lateral size the modification of FESEM of analysis has been done with Imag-J software. |

| 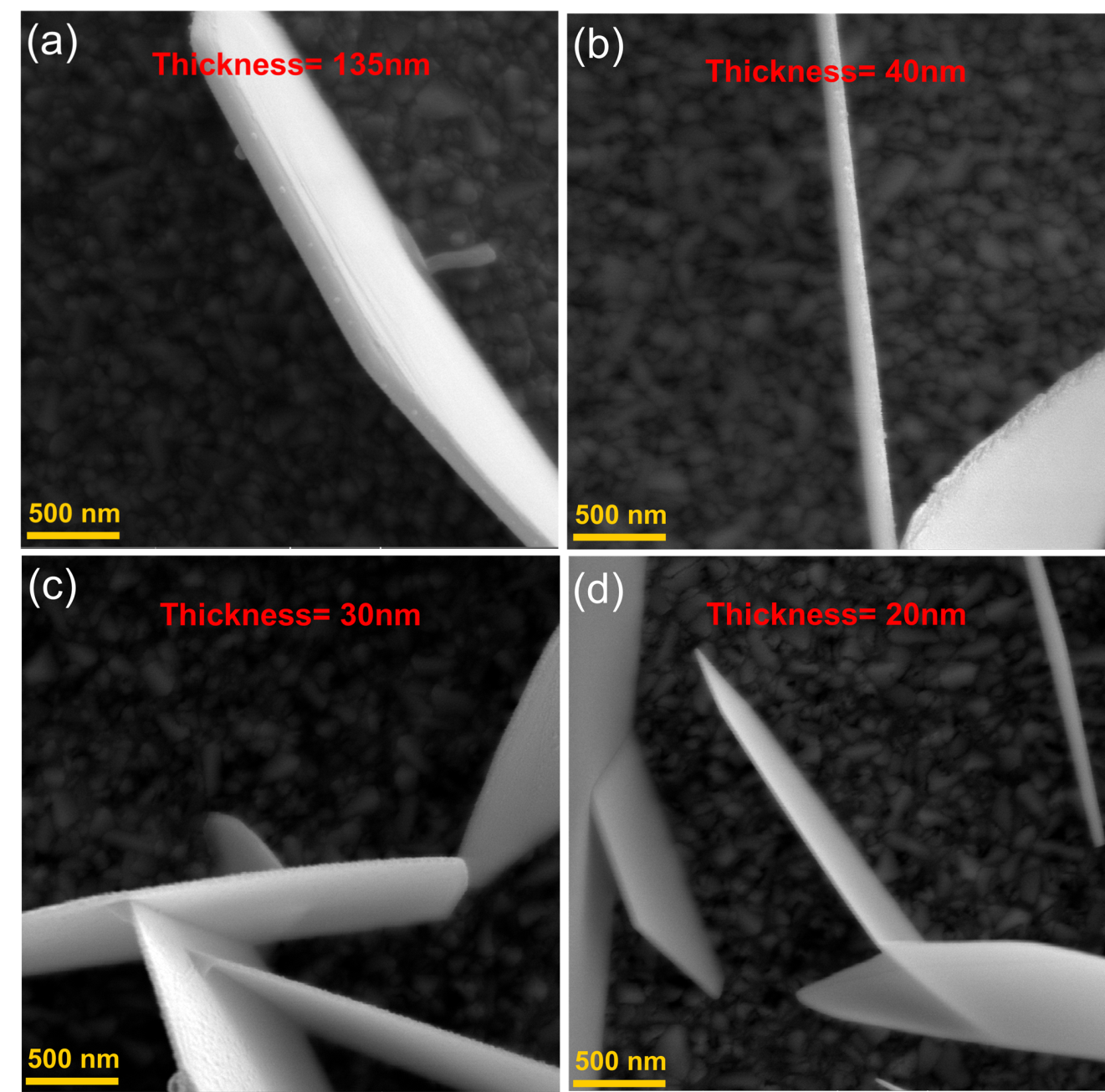 |
| --- |
| Fig. S3. The influence of sulfur amount on the thickness of SnS_2_ nanosheets.  FESEM analysis of vertically grown SnS_2_ nanosheets on FTO substrate through modified CVD method, by using different amount of sulfur of a)200mg, b)300mg, c)400mg and d)500mg. To calculate the average thickness of the SnS_2_ flakes, the high magnification of FESEM analysis has been performed |

**Fig. S4. The Wulff’s model on morphology of SnS_2_ nanosheet.**

For a free crystal growing near the equilibrium condition, the crystal shape for different λ (different sizes) is self-similar around the Wulff’s point. For a supported crystal “A” on substrate “B”, the equilibrium shapes were determined by minimization of surface and interface energies. For the case of a strained polyhedral crystal, A supported on substrate B, similar to SnS_2_ nanosheet growth in this study, elastic bulk energy plays an important role

| 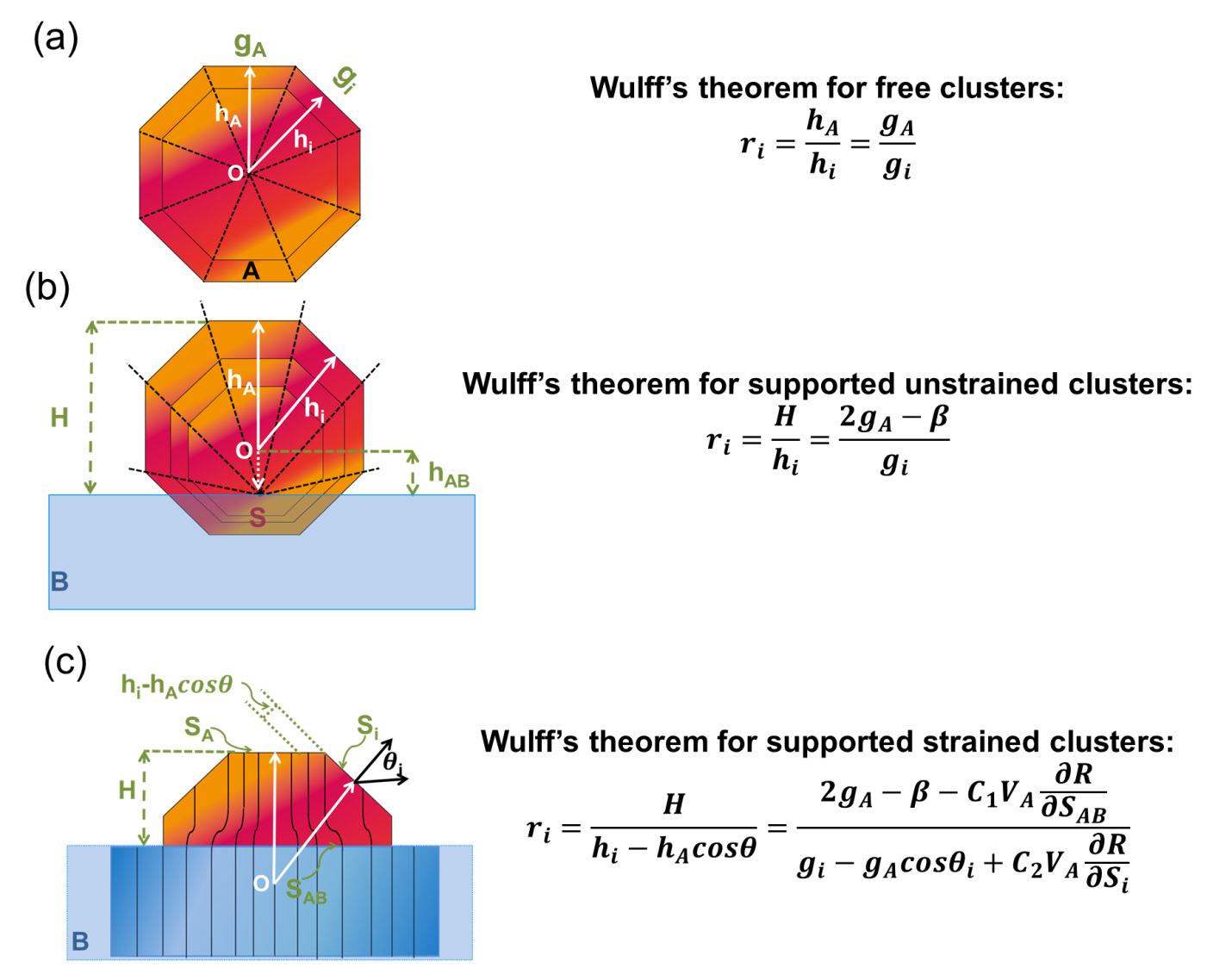 |
| --- |
| Fig. S4. Wulff’s theory.  Schematic of (a)Free crystal and (b)As-deposited crystal with their related constancy of shape ratio^44^. |

**Fig. S5. Photocurrent and optical results:** The novel fabricated photodetector based SnS_2_ nanosheets, provide the illumination from glass side as shown in the Fig. S5a. The [logarithmic current](https://www.google.com/search?sxsrf=ALeKk00J0zyFnHyHV90K1dJpwAhFSG6NzA:1599292121848&q=logarithm+current&spell=1&sa=X&ved=2ahUKEwiZ8sG2w9HrAhVBCewKHWycB-0QkeECKAB6BAgNECU) versus applied voltage provides the highest current under the wavelength of 450 nm illumination (at the same intensity of 5mW/cm^2^ and -3V bias voltage) as shown in Fig. S5b. The photocurrent is indicating Schottky like junction at FTO-SnS_2_ contacts, due to junction of FTO with a higher work function (−4.4eV) in contact with the SnS_2_ semiconductor layer with lower conduction energy level (−4.2) as shown in the inset of Fig. S5b. The stability of the photodetector based pristine grown SnS_2_ versus time has been investigated under illumination/dark exposure modes for a long time at 1Hz and 1kHz frequency at 445 nm wavelength and 3V bias as shown in Fig. S5c and Fig. S5d, respectively

| 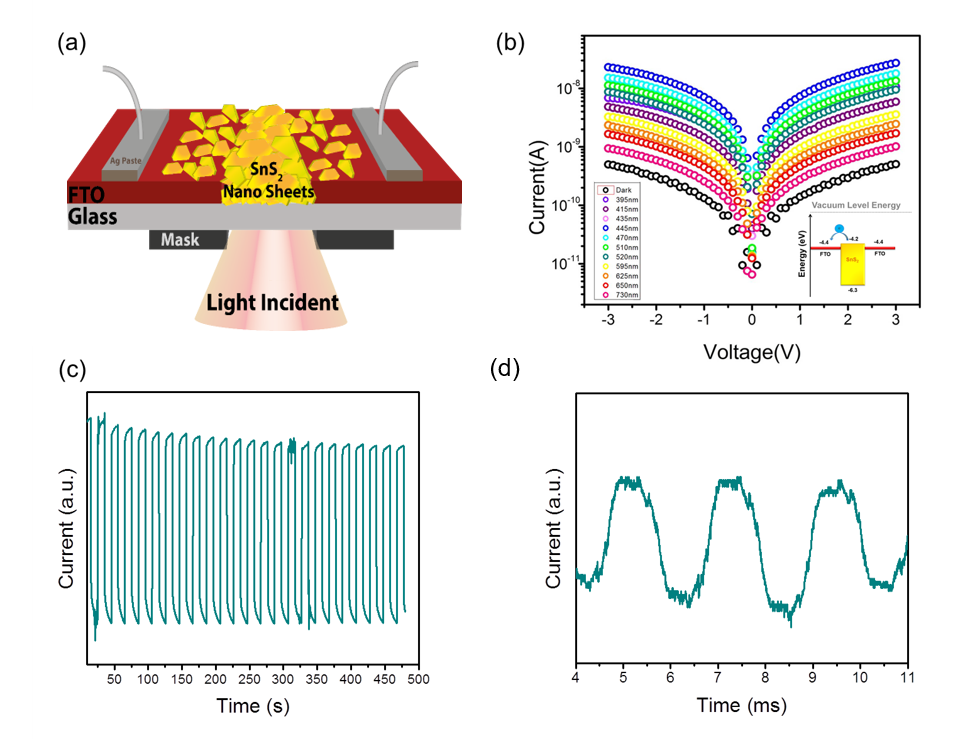 |
| --- |
| Fig. S5. SnS_2_ pristine based photodetector.  (a) Schematic of fabricated photodetector based on SnS_2_ nanosheets. (b). The semi-log of current evolution versus biased voltage of photodetector SnS_2_ for different illuminated wavelengths, at same light intensity of 5mW/cm^2^. The inset shows the energy band alignment of FTO/SnS_2_/FTO Schottky junction. I-t curves for SnS_2_ based photodetector at (c) f=1Hz and (d) f=1kHz at 3V bias, under 445 nm wavelength for several times |

**Fig. S6. TCLP characterization**

In Fig. S6 the XRD and optical characterization of TCLP layer has been shown. The crystalline structure of perovskite layer is illustrated in Fig. S6a. The diffraction peaks at 14.12 o, 20.00 o, 24.51 o, 28.38 o, 31.80 o, 40.53 o and 43.12o correspond to the crystalline planes of photo active black phase of Cs0.05 (MA0.17 FA0.83)0.95 Pb (I0.83 Br0.17)3 perovskite layer; the peaks related to TCLP and substrate are indicated by * and O, respectively. The absorption spectra and PL measurement of the perovskite layer is presented in Fig. S6b.


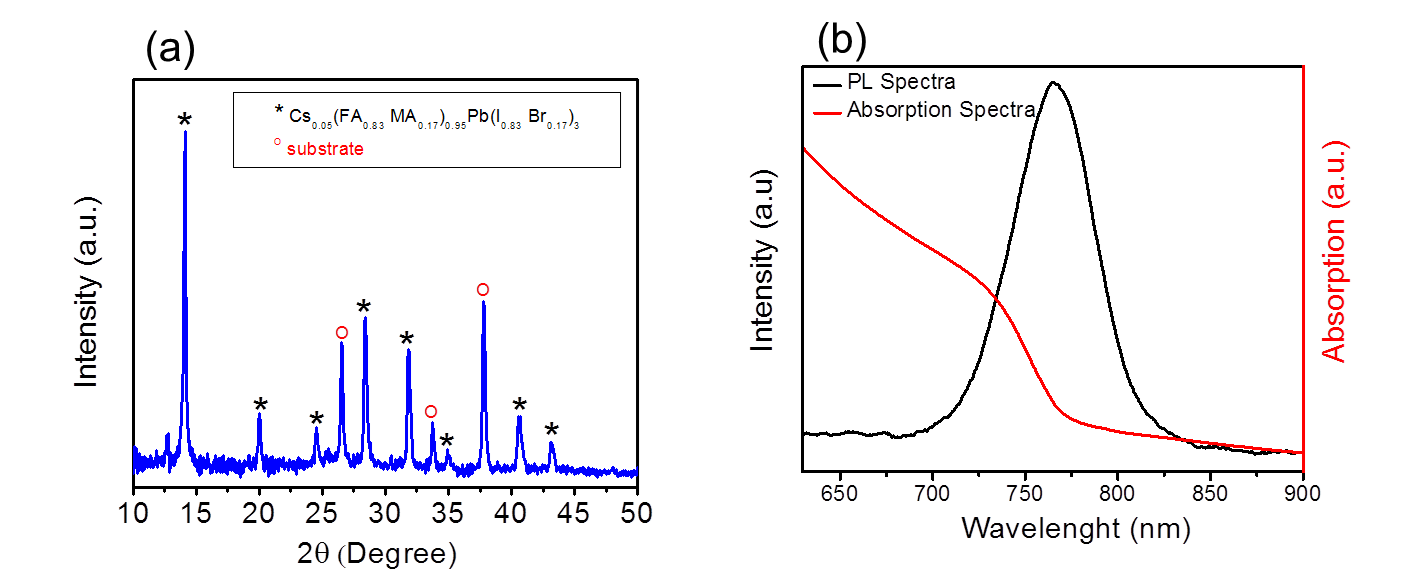


Fig. S6. Cs_0.05_(FA_0.83_MA_0.17_)_0.95_Pb(I_0.83_Br_0.17_)_3_ layer characterization.

(a) XRD pattern and (b) optical analysis including absorption spectra and photoluminescence of the pure TCLP film

**Fig. S7. SnS_2_/TCLP photodetector characterization**

| In Fig. S7, the thickness of SnS_2_/ TCLP which is about 5.5µm by FESEM analysis (Fig.S7a) is almost same to the thickness of pristine grown SnS_2_ nanosheets on the FTO (as shown in Fig.. 1C –inset). The absorption spectrum versus several wavelength of 350nm to 1000 nm has been investigated for pristine SnS_2_ nanosheets, pristine TCLP layer and the SnS_2_/ TCLP heterojunction, as shown in Fig. S7b. In Fig. S7c the logarithmic current versus applied voltage for SnS_2_/TCLP hybrid photodetector shows the enhancement of the current at the wavelength of 445 nm in compare with the other incident wavelengths. The stability of the SnS_2_/TCLP photodetector versus time has been investigated under illumination/dark exposure modes for a long time at 1Hz and 2 kHz frequency at 445 nm wavelength and 3V bias as shown in Fig.S7d and Fig.S7e, respectively. The current-voltage behavior has been investigated for pristine TCLP perovskite photodetector as the control sample (Fig. S7f).  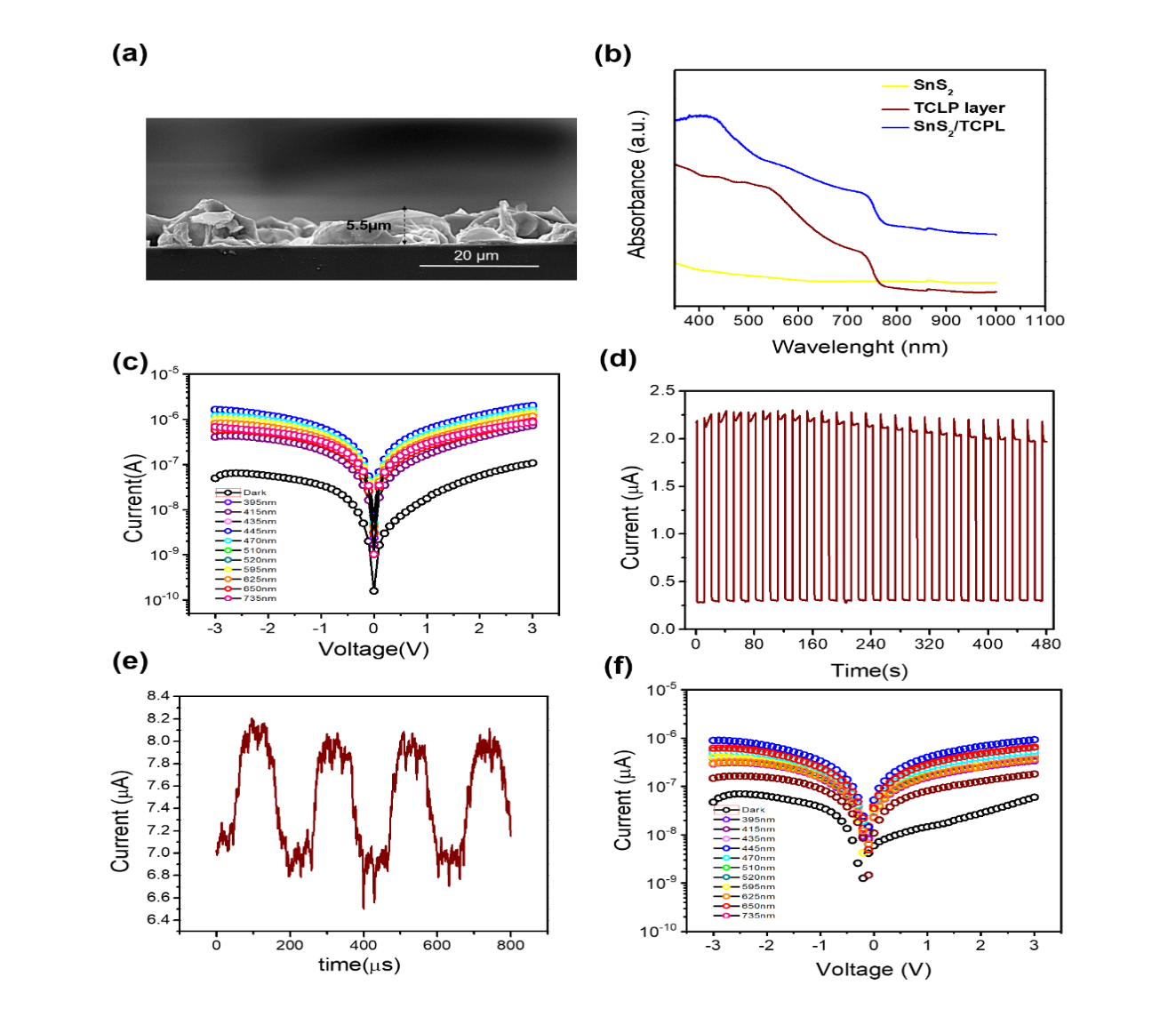 |
| --- |
| 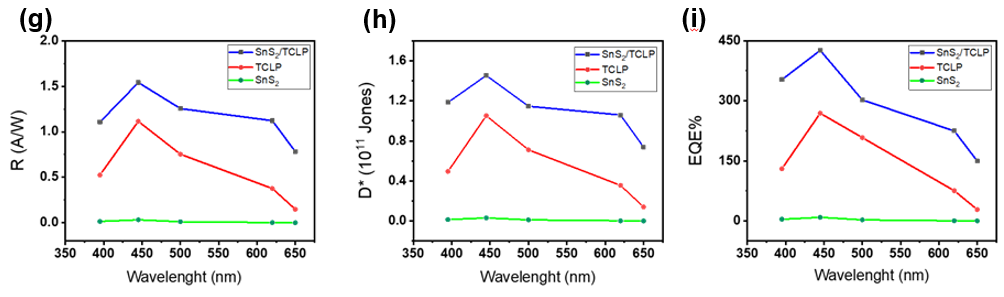  Fig. S7. SnS_2_/TCLP based photodetector characterization.  (a). FESEM analysis of SnS_2_/TCLP cross section. (b) Absorption graph of pristine SnS_2_, pristine TCPL layer and SnS_2_/TCLP (c) The semi-log of current evolution versus biased voltage of SnS_2_/TCLP PD for different illuminated wavelengths at same light intensity of 5mW/cm^2^. (d) I-t curves for SnS_2_/TCLP heterojunction photodetector at f=1Hz and (e) f=2kHz under 3V bias for 445 nm wavelength cyclic illuminations. (f) The semi-log of current evolution versus biased voltage of pure TCLP device for different illuminated wavelengths at same light intensity of 5mW/cm^2^. (g-i) Photodetector parameters for three fabricated SnS_2_, SnS_2_/TCLP and pure TCLP based devices under different illuminated wavelength and equal intensity of 5mW/cm^2^. |

**Fig. S8. Protective PMMA layer**. Using the protecting layer of 20%wt PMMA has been used to improve the stability of photodetector under the 100% humidity and the room temperature. The change in the appearance of the protected and un protected device after storing for 10 minutes has been provided in Fig.S6 show the yellowish colour of TCLP layer, which indicated the decomposition of Pb-I.

| 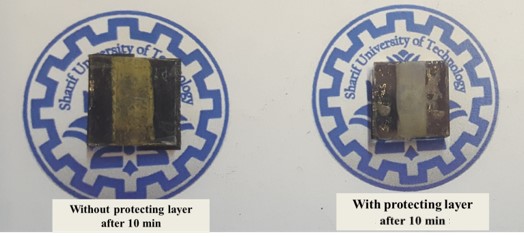 |
| --- |
|  |

Fig. S8. The picture of the SnS2/TCLP with and without PMMA as the protecting layer after 10 min under 100% humidity situation

**S9. The schematic of SnS_2_/TCLP photodetector fabrication process:** To achieve the SnS_2_/TCPL photodetector, laser engraved performed on the FTO/glass substrate and a ~25µm gap on the FTO substrate created. This substrate was used in CVD process along with 500mg of sulfur element. After growing the SnS2 nanosheets on the substrate, the TCLP precursor has been deposited on the SnS2 nanosheets through two-step spin-coating process at 1000 rpm and 4000 rpm for 10 s and 30 s, respectively. The film was then baked at 100ºC for 1 hour. Finally, the incident light illuminated from backside of the fabricated PD as shown in Fig. S10 g)

| 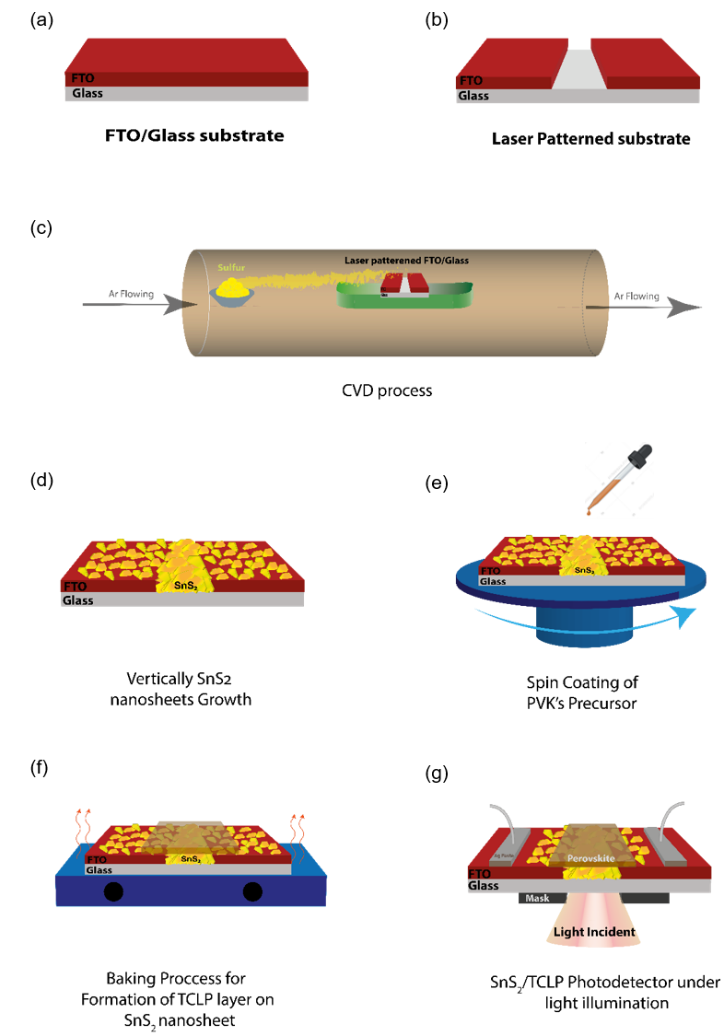 |
| --- |
| Fig. S9- the schematic of SnS_2_/TCLP PD fabrication. (a) FTO/glass (b) Patterned laser FTO/glass with the 25µm strip on it (c) the CVD process by utilizing the sulfur amount of 500mg (d)The vertically SnS_2_ nanosheets growth on the FTO/glass groove (e ) the spin-coating deposition of TCLP precursor on the SnS_2_ nanosheets (f) baking process of the TCLP deposition at 100 °C for 1 h. (g) The addition SnS_2_ has been removed physically and then Ag paste has been used for better electrical of the SnS_2_/TCLP PD, the mask with the effective area of the 1.36E-6 cm^2^ has been installed on the back side of the PD and finally the light has been illuminated from back side of it. |

**S10. Rising/falling time circuit measurement:** To measure the rising and falling time of the photodetector, an operational amplifier circuit and an oscilloscope have been utilized. A pulsed light has been used controlled by GWINSTEK GDS-1052-U oscilloscope along with a current to voltage converter circuit

| 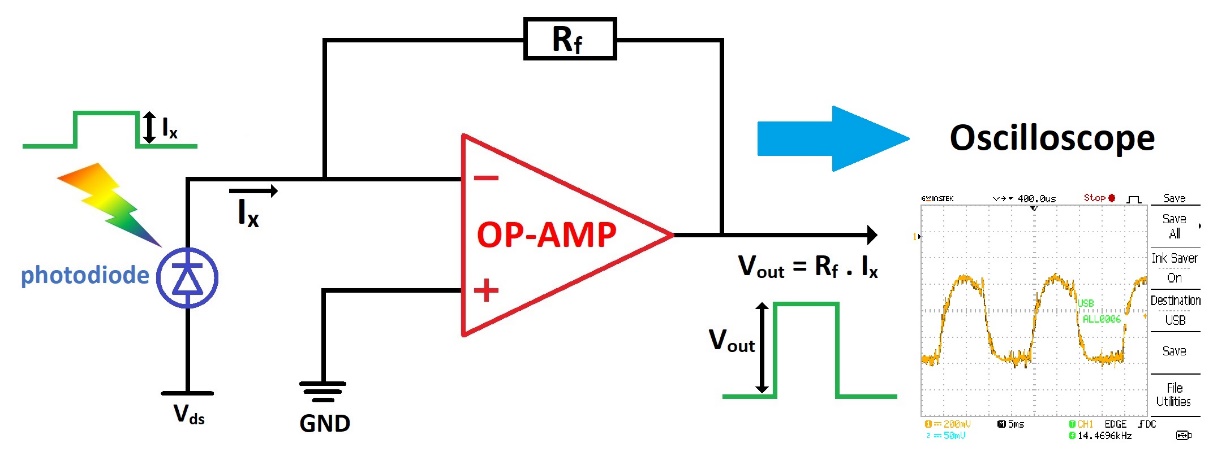 |
| --- |
| Fig. S10. The circuit measurement of the rising/falling time of the PD in this research. The amount of the current in the dark and light has been calculated via the amount the out-put voltage and the resistance in the circuit |
